# GLIMS: A two-stage gradual-learning method for cancer genes prediction using multi-omics data and co-splicing network

**DOI:** 10.1016/j.isci.2024.109387

**Published:** 2024-03-02

**Authors:** Rui Niu, Yang Guo, Xuequn Shang

**Affiliations:** 1 School of Computer Science, Northwestern Polytechnical University, Xi’an 710129, China; 2 School of Information Science and Engineering, Lanzhou University, Lanzhou 730000, China

**Keywords:** Biocomputational method, Cancer systems biology, Cancer, Omics, Machine learning

## Abstract

Identifying cancer genes is vital for cancer diagnosis and treatment. However, because of the complexity of cancer occurrence and limited cancer genes knowledge, it is hard to identify cancer genes accurately using only a few omics data, and the overall performance of existing methods is being called for further improvement. Here, we introduce a two-stage gradual-learning strategy GLIMS to predict cancer genes using integrative features from multi-omics data. Firstly, it uses a semi-supervised hierarchical graph neural network to predict the initial candidate cancer genes by integrating multi-omics data and protein-protein interaction (PPI) network. Then, it uses an unsupervised approach to further optimize the initial prediction by integrating the co-splicing network in post-transcriptional regulation, which plays an important role in cancer development. Systematic experiments on multi-omics cancer data demonstrated that GLIMS outperforms the state-of-the-art methods for the identification of cancer genes and it could be a useful tool to help advance cancer analysis.

## Highlights

• GLIMS is a two-stage gradual-learning method to identify cancer genes accurately
• HIM-GCN model can integrate omics data and PPI network to identify cancer genes
• Integrating cancer-specific co-splicing network can optimize initial prediction

## Graphical abstract

**Figure:**
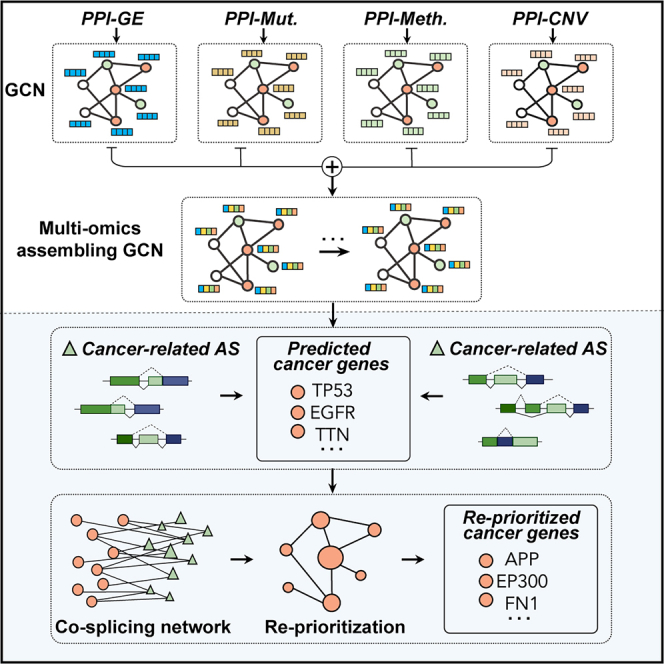

## Introduction

Cancer is a type of disease driven by genomic aberrations and has become one of the greatest threats to human health nowadays. The identification of cancer genes is crucial for the diagnoses and therapies, especially for precision oncology.^1^^,^^2^ The rapid development of high-throughput sequencing has generated a huge amount of cancer genomic data from multiple omics studies such as The Cancer Genome Atlas (TCGA)^3^ databases and provides exciting opportunities to study cancer using computational methodologies. Based on cancer genomic sequencing data, various cancer studies have been performed and novel cancer knowledge has been constantly revealed in recent years. However, as a complete biological process always accompanies various gene regulations or chain-reactions in multi-omics views, identifying cancer genes is quite complex. A considerable challenge arises because most gene aberrations or abnormal regulations act as passenger casual elements and only a few of them play driver roles in cancer occurrence and progression. It is difficult to identify cancer genes from a large set of genes without additional information. In addition, cancer occurrence is also affected by abnormal gene regulations at the multi-omics level, such as gene transcriptome and methylation regulations. Therefore, it is essential to develop methods integrating multi-omics data to improve the prediction accuracy of cancer genes.

In the past decade, many methods have been developed for the identification of cancer genes, mainly including two branches in terms of supervised and unsupervised manners. The supervised methods first train a prediction model based on limited knowledge of known cancer genes and then use the learned model to predict new cancer genes. For instance, the 20/20+^4^ method uses the 20/20 rules to learn a random forest model to predict cancer driver mutations and genes. MoProEmbedding^5^ applies a network-based propagation approach to learn gene embeddings and predict novel cancer genes. EMOGI^6^ integrates multi-omics data and PPI network to predict cancer genes using a semi-supervised learning approach. MTGCN^7^ uses the multi-task learning approach to identify cancer driver genes by augmenting gene features with the PPI network. Additionally, MODIG^8^ employs a graph attention network to model interactions within dimensions in multi-dimensional gene networks and integrates joint learning to generate representations of genes. Although these methods can meet some of the needs of cancer gene analysis to some extent, high-quality prior knowledge of cancer genes is required to obtain a better prediction model. In contrast, the unsupervised methods directly predict cancer genes without prior knowledge of the cancer genes, such as MutSig2CV,^1^^,^^9^ HotNet2,^10^ NetICS,^11^ and Moonlight.^12^ MutSig2CV calculates the significance of gene mutations in cancer samples to predict cancer genes directly; HotNet2 detects the cancer gene sets by incorporating gene networks; NetICS applies a network diffusion method to prioritize cancer genes by integrating multi-omics data and PPI network; Moonlight identifies context-dependent cancer genes by integrating cancer-related biological processes from the literature and gene-gene interactions in transcriptomic data. Unsupervised methods operate independently of well-established knowledge regarding cancer genes, but their efficacy heavily depends on the quality of data. Despite the numerous computational methods available, several challenges need to be further focused. First of all, the integration ability to consider more data types still needs to be extended, since cancer is usually induced by abnormal regulations at multi-omics levels, such as DNA mutation,^13^ RNA alternative splicing (AS),^14^^,^^15^^,^^16^^,^^17^ and protein regulation.^18^ In addition, the overall estimation of cancer genes also needs to be improved to satisfy the demands of cancer-related analysis.

**Figure 1:**
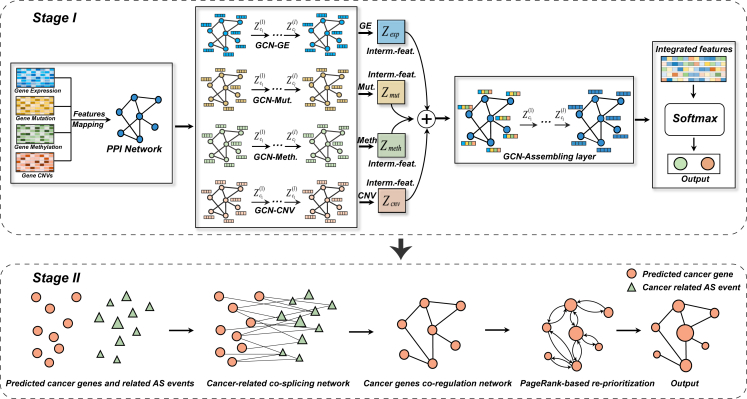
Schematic of the GLIMS framework Stage I: predicting candidate cancer genes using the HIM-GCN model. The PPI network is integrated with four types of multi-omics data to form attributed gene association networks. Single-layer GCN models are then employed to capture gene representations from each network; these are concatenated and further refined by a two-layer GCN to clarify genes into predicted cancer genes and non-cancer genes based on their output probabilities. Stage II: enhancing the initial prediction outcomes through co-splicing network optimization. HIM-GCN-predicted cancer genes (orange circles) and cancer-related AS events (green triangles, sized by PSI value) are used to construct the cancer-related co-splicing network using partial correlation coefficients. Through the examination of shared AS targets, this process leads to a co-regulation network. This enhanced network comprises cancer genes that are subsequently re-prioritized using the PageRank algorithm, with the post-optimization probabilities then reflected in the resized orange circles.

In this paper, we present a two-stage gradual learning framework to identify cancer genes by integrating multi-omics data and co-splicing network, which is noted as GLIMS (see Figure 1). In the first stage, a hierarchical integration model based on graph convolutional network, named HIM-GCN, was developed to predict initial candidates of cancer genes through semi-supervised learning based on limited knowledge of known cancer genes. In the next stage, we further optimized the initial prediction outcomes by incorporating the information of AS regulations using unsupervised learning.^19^^,^^20^ The advantages of GLIMS are as follows: (1) it uses a two-stage gradual-learning strategy to improve the identification of cancer genes in an integral learning system; (2) the proposed semi-supervised HIM-GCN model inherently integrates multi-omics features and relationships between genes to predict cancer genes according to an end-to-end hierarchical learning structure, which considers the imbalanced learning of few known cancer genes; (3) it incorporates AS regulation to improve the identification of cancer genes.

## Results

### The effectiveness studies of the prediction model HIM-GCN

#### Performance comparison studies in identifying cancer genes

To evaluate the prediction performance of the proposed HIM-GCN model in GLIMS, we first conducted comparison studies on multiple cancer datasets. Specifically, we compared HIM-GCN with the existing popular methods toward the identification of cancer genes, including HotNet2,^10^ MutSig2CV,^1^ MoProEmbedding,^5^ EMOGI,^6^ MODIG,^8^ and MTGCN.^7^ Additionally, we designed a DeepWalk-based model and an original graph convolutional network (GCN) model for comparison.^21^^,^^22^ The DeepWalk-based model employs a random deep walking algorithm to extract features from the PPI network. These features are then combined with multi-omics features to form gene representations, which are subsequently fed into a support vector machine (SVM) classifier. The original GCN method uses graph neural network to learn gene representations in PPI network without omics features and predict cancer genes in a semi-supervised manner. We predicted cancer genes in Pan-cancer and three cancer datasets, breast cancer (BRCA), glioblastoma (GBM), and lung adenocarcinoma (LUAD), comparing their performance on each on test dataset using multiple measurements. 3-fold cross-validation was used to guide the training process, and independent test datasets derived from known labels were used to evaluate the performance of each method systematically. As shown in Table 1, the proposed HIM-GCN model consistently outperforms other existing methods in the Pan-cancer dataset. Meanwhile, as shown in Table 2, HIM-GCN achieves better performance on most measurements in the three specific cancer datasets. The results demonstrate that HIM-GCN has a strong ability to predict cancer genes in more accurate anticipation by integrating multi-omics data according to the hierarchical graph deep learning framework.

**Table 1:** The performance of different prediction methods on the Pan-cancer dataset

Methods	Accuracy	AUROC	AUPRC	F1-score
DeepWalk+SVM	0.727	0.756	0.617	0.549
EMOGI	0.793	0.773	0.571	0.521
GCN	0.557	0.499	0.364	0.271
HotNet2	0.789	0.623	0.495	0.176
HIM-GCN	**0.858**	**0.904**	0.768	**0.667**
MODIG	0.751	0.822	0.592	0.617
MoProEmbedding	0.654	0.718	0.645	0.549
MutSig2CV	0.773	0.595	0.397	0.169
MTGCN	0.834	0.846	**0.792**	0.633

**Table 2:** The performance of different prediction methods on BRCA, GBM, and LUAD datasets

Methods	BRCA				GBM				LUAD			
	Accuracy	AUROC	AUPRC	F1-score	Accuracy	AUROC	AUPRC	F1-score	Accuracy	AUROC	AUPRC	F1-score
DeepWalk+SVM	0.832	0.789	0.514	0.428	0.764	0.667	0.401	0.316	0.843	0.683	0.411	0.369
EMOGI	0.796	0.902	0.652	0.386	0.676	0.917	0.573	0.313	0.838	0.315	0.138	0.241
GCN	0.717	0.569	0.290	0.202	0.644	0.497	0.235	0.140	0.704	0.514	0.221	0.143
HotNet2	0.826	0.724	0.385	0.150	0.890	0.492	0.264	0.139	0.925	0.636	0.326	0.167
HIM-GCN	**0.924**	**0.932**	0.645	0.583	0.865	**0.928**	**0.641**	**0.541**	0.909	**0.814**	**0.511**	**0.539**
MODIG	0.798	0.861	0.523	0.529	0.687	0.633	0.219	0.195	0.786	0.552	0.315	0.270
MoProEmbedding	0.884	0.785	0.612	0.516	0.630	0.723	0.530	0.313	0.554	0.714	0.504	0.381
MutSig2CV	0.920	0.798	0.570	0.389	**0.912**	0.721	0.594	0.353	**0.932**	0.743	0.322	0.267
MTGCN	0.901	0.889	**0.684**	0.576	0.893	0.885	0.638	0.532	0.915	0.804	0.510	0.457

#### Disease enrichment analysis of the predicted cancer genes

**Figure 2:**
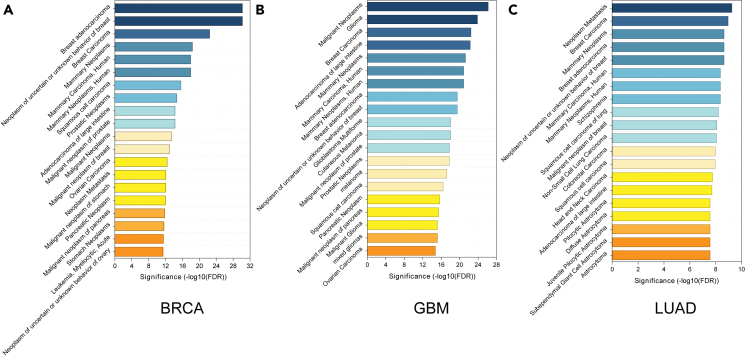
The DisGeNET analysis revealed the top 20 enriched disease terms associated with cancer genes identified by HIM-GCN (A–C) The top 20 enriched disease terms in BRCA, GBM, and LUAD, respectively.

To further explore the ability of HIM-GCN in predicting cancer type-specific genes, we conducted disease enrichment analysis of the predicted cancer genes in three cancer types. Here, we first obtained candidate cancer genes with possibilities > 0.7 in HIM-GCN and obtained 417, 485, and 387 candidate cancer genes in BRCA, GBM, and LUAD, respectively. The disease enrichment analyses were performed by DisGeNET^23^ (Figure 2). In the BRCA dataset, most of the top enriched disease terms are related to breast cancer, such as "Breast Adenocarcinoma" (false discovery rate (FDR) = 6.63e−31), "Breast Carcinoma" (FDR = 3.47e−23), etc. In GBM and LUAD datasets, we can observe similar situations where most of the top enriched terms are also related to the corresponding disease types, although some of them may not be the most significant ones. In addition, we also observe that there exist overlaps between the enriched disease lists in three cancers. This may be because there are some basic common cancer genes that involved in similar cancer diseases. In conclusion, the results of disease enrichment analysis further demonstrate that our proposed HIM-GCN model predicts more meaningful cancer genes in terms of interpreting cancer occurring at functional levels.

#### Effectiveness studies in helping cancer subtyping

It is well known that most cancers include multiple subtypes with different biomarkers and abnormal regulation mechanisms. As we have demonstrated that the predicted cancer genes from HIM-GCN are closely related to the occurrence of corresponding cancers and can be deemed as their biomarkers in raising cancer disease, we further examine the effectiveness of the predicted cancer genes in cancer subtyping, as well as their clinical survival differentials. Many studies proved that gene expression and methylation information play important roles in cancer.^24^^,^^25^^,^^26^ Therefore, we used the popular similarity network fusion^27^ method on gene expression and methylation data of predicted biomarker genes to identify different subtypes. To understand the specific linked functions of the predicted biomarker genes in recognizing cancer subtypes, we divided those biomarker genes into different clusters according to their gene expression features by using clustering algorithm and then conducted gene ontology (GO) as well as pathway enrichment analyses for those gene clusters.

**Figure 3:**
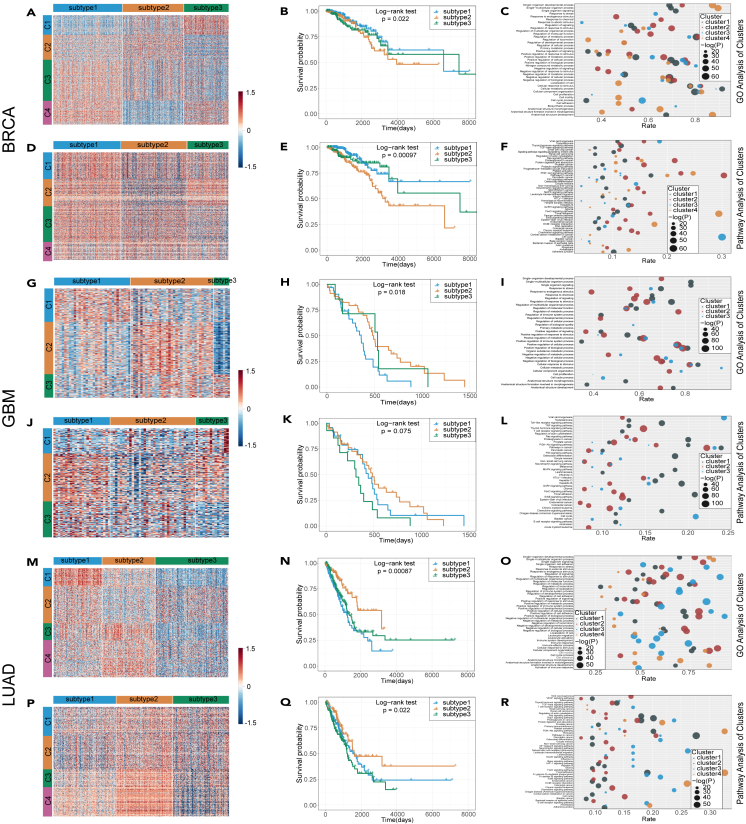
Cancer subtying analyses The predicted candidate cancer genes play significant roles in cancer subtyping, as illustrated by the heatmaps using gene expression data (A, G, M), and methylation data (D, J, P). Survival analyses of identified cancer subtypes derived from gene expression data (B, H, N), as well as methylation data (E, K, Q), statistical siginificance is defined as p < 0.05. Enrichment analyses of these genes within GO (BP) (C, I, O) and pathway (F, L, R).

Figure 3 presents a comprehensive overview of the cancer subtyping analyses performed on three cancer datasets. As shown in Figures 3A and 3D, when utilizing solely the predicted cancer genes as features in the BRCA, distinct cancer subtypes were identified on both gene expression and methylation data. To evaluate the clinical relevance of these subtypes, we then conducted survival analyses. Significant differential survival patterns among the subtypes were identified based on gene expression data (Figure 3B, p=2.2e−2) and methylation data (Figure 3E, p=9.7e−4). These findings highlight the utility of the predicted biomarker genes in discovering more clinically meaningful subtypes in breast cancer. Furthermore, we investigated the interrelated functions of these genes in cancer development and subtyping. GO and pathway enrichment analyses were performed using the DAVID tool,^28^^,^^29^^,^^30^^,^^31^ focusing on gene clusters from expression patterns. Most gene clusters are enriched in signaling regulation, cell-cycle processes, and other relevant terms within the biological process ontologies, as shown in Figure 3C, which are closely associated with the metabolic process in breast cancer development. Additionally, we observed that the most enriched pathways were closely linked to specific cancer diseases and related signaling regulation pathways (Figure 3F). Using the same methods, we analyzed the predicted cancer genes in GBM and LUAD datasets. In GBM, three distinct subtypes were identified (Figures 3G and 3J), which exhibited statistically significant differences in survival patterns (Figures 3H and 3K), and the enriched biological functions and pathways were found to be cancer related (Figures 3I and 3L). For LUAD, we obtained similar results with the predicted cancer genes in cancer subtyping, and their functions were also enriched in cancer-related biological processes and pathways (Figures 3M–3R). In summary, our HIM-GCN model accurately predicts cancer biomarker genes and cancer subtypes and facilitates the interpretation of the underlying mechanisms driving cancer occurrence and development.

#### Case study of survival effects

**Figure 4:**
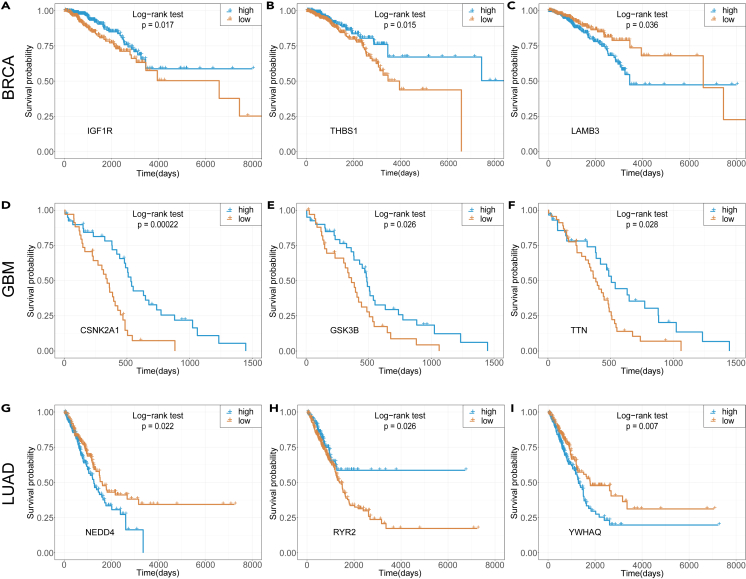
Predicted cancer genes function as biomarkers in predicting patient survival outcomes (A–I) For each cancer type, the top-3 biomarker genes are selected from the pool of predicted cancer genes. Patients are stratified into “high” and “low” groups based on the average gene expression levels of each selected gene, statistical significance is defined as p < 0.05.

We hypothesized that cancer biomarker genes play a crucial role in the biological regulation of cancer progression, and their expression levels may provide insights into the estimation of clinical survival time. In our study, we selected the top 3 biomarker genes predicted by HIM-GCN, none of which were previously listed as known cancer genes, to investigate the survival trends of patient samples with different expression levels in each cancer dataset. For each biomarker gene, we divided all patient samples into “high” and “low” expression groups based on average expression level across all samples. In the BRCA dataset, the biomarkers exhibited significant differential survival trends (Figures 4A–4C). Among them, IGF1R and THBS1 are identified as potential cancer genes in the Network of Cancer Genes (NCG)^32^ dataset, while LAMB3 is a newly predicted biomarker gene for breast cancer from our predictions. LAMB3 has been previously studied as a tumor-related gene and is known to mediate cell-cycle arrest and apoptosis.^33^ In the GBM dataset (Figures 4D–4F), CSNK2A1 and TTN are similarly identified as potential cancer genes as in NCG.^32^ Our study identifies GSK3B as a novel glioblastoma cancer gene, and it has been demonstrated to be aberrantly activated in various cancer types, promoting tumor cell proliferation and immortalization.^34^ For LUAD, as shown in Figures 4G–4I, both NEDD4 and RYR2 are marked as potential cancer genes in NCG, while YWHAQ is a new biomarker for lung cancer identified by HIM-GCN. In conclusion, our case studies suggest that the HIM-GCN-predicted cancer genes could provide valuable clinical insights into cancer prognosis, particularly for survival estimation based on gene expression levels.

### The effectiveness studies of using co-splicing network to improve prediction outcomes

#### AS provides useful information for cancer recognition

**Figure 5:**
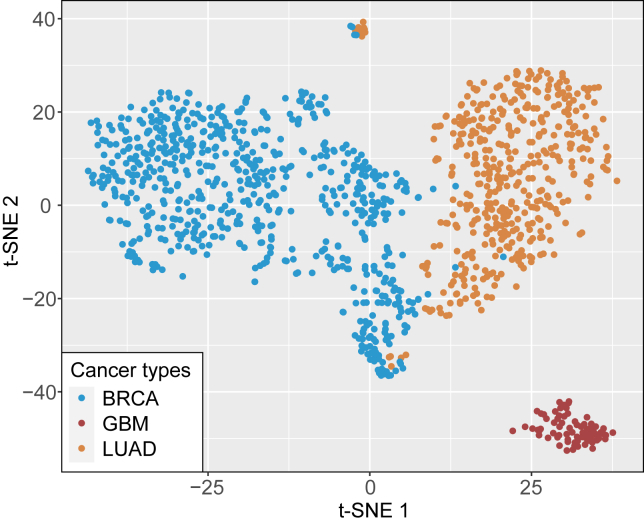
The scatterplot of different cancer types based on their common alternative splicing events

Numerous studies have demonstrated the important role of pre-mRNA AS in post-transcriptional regulations of cancer.^15^^,^^18^^,^^19^ Accordingly, we investigated how integrating pre-mRNA AS information into the GLIMS model could enhance the prediction accuracy of cancer genes based on HIM-GCN outputs. We selected informative AS events by applying three filtering conditions: (1) an average percentage spliced in index (PSI) value across samples > 0.1; (2) a variance in PSI across samples > 0.02; and (3) an absolute $\log_{2}\left(\text{fold}-\text{change}\right)$ in PSI between normal and tumor samples > 0. We then utilized the PSI features of all common AS events across the three cancers to distinguish cancer types in a sample mixture condition. As shown in Figure 5, significant differential AS patterns were observed in the three cancer types.

**Figure 6:**
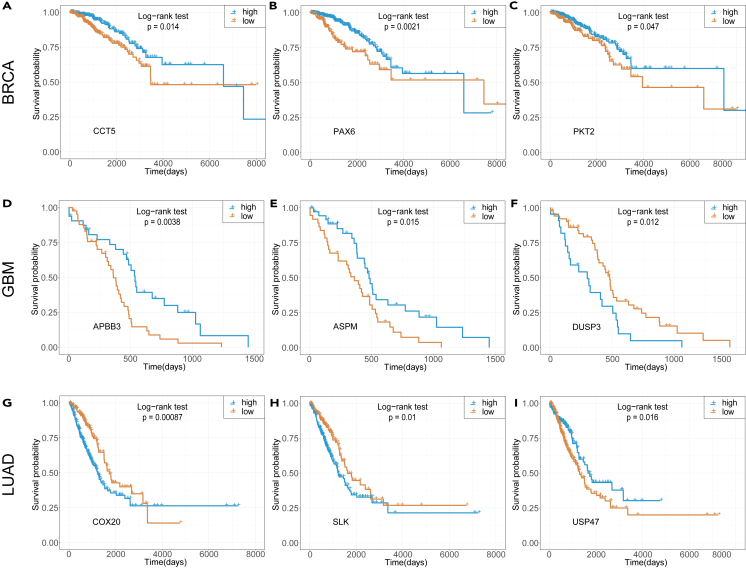
Alternative splicing events have utility as prognostic biomarkers in predicting patient survival outcomes (A–I) In each cancer type, three SE events exhibiting differential expression between cancer and normal tissues are selected. The patients were divided into “high” and “low” groups based on the average PSI values associated with each event. statistical significance is defined as p < 0.05.

We further investigated the impact of AS events on clinical survival phenotype. We conducted case studies to analyze the survival outcomes associated with skipping exon (SE) events across three cancer types. For each cancer type, we selected three cases for which the differential analysis between cancer and normal samples showed significant changes ($|\log_{2}\left(fold-change\right)|>1.5$). We then performed survival analysis on all cases, where samples were stratified into two groups (“high” and “low”) based on the average PSI level of each case. As shown in Figures 6A–6I, each of the investigated SE events exhibited significant differential survival trends. Our findings indicate the importance of AS regulation in cancer recognition and clinical prognosis and suggest the potential of incorporating AS data to enhance cancer gene prediction.

#### Effectiveness study of using co-splicing network to improve cancer gene prediction

**Figure 7:**
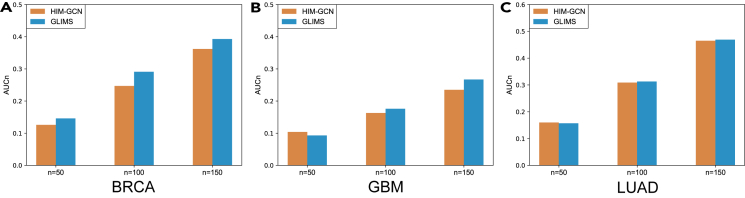
Effectiveness studies of utilizing cancer-related co-splicing networks (A–C) The AUC_n_ of BRCA, GBM, and LUAD, respectively.

In the previous section, we evaluated the fact that the AS regulation provides informative clues for cancer recognition and clinical survival estimation. To evaluate the effectiveness of co-splicing network in enhancing cancer gene estimation, we compared the predictive performance of cancer genes before and after AS-based optimization by using the partial receiver operating characteristic (ROC) measure (as shown in Figure 7). For BRCA and GBM, the utilization of co-splicing networks led to improved performance in terms of $AUC_{n}$, emphasizing its ability to refine the prediction of cancer-specific genes. However, in LUAD, only a slight performance improvement was observed with co-splicing network optimization. This may be due to the relatively minor impact that AS regulation has on the overall regulatory processes in lung cancer. In the second step of the GLIMS process, the co-splicing network could potentially optimize the overall performance of cancer gene prediction in most datasets.

#### Case study of predicted cancer genes attends cancer-related AS regulation pathway

Having demonstrated the important role of AS regulation in improving the identification of cancer genes, we further conducted case studies on the regulatory pathway between the predicted cancer genes and cancer-related AS events. RNA-binding proteins (RBPs), also known as splicing factors (SFs), are proteins that can bind RNA splicing sites and regulate specific RNA AS events. We hypothesized that the predicted cancer genes may influence SFs and thereby regulate cancer-related AS events in the cancer metabolic pathway. To verify the hypothesis that the predicted cancer genes are involved in cancer-related AS regulation pathways, we constructed a tripartite network consisting of the predicted cancer genes (CGs), cancer-related SFs, and cancer-related AS events in each cancer dataset. Given that genes within the same expression clusters frequently exhibit similar biological functions, we used the cancer gene clusters based on expression data (Figures 3A, 3G, and 3M) as the fundamental units. Within each cancer gene cluster, we initially identified the cancer-related SFs by referencing a list of RBPs.^35^ Then we constructed a tripartite network comprising CG-SF-AS relationships (focusing on SE events) by performing correlation analysis among the different elements. The CG-SF connections were retained if their absolute correlation coefficients exceeded the specified threshold (BRCA: ≥0.65; GBM: ≥0.75; LUAD: ≥0.5). Similarly, we determined SF-AS connections based on absolute partial correlation coefficients (BRCA: ≥0.4, GBM: ≥0.35; LUAD: ≥0.2).

**Figure 8:**
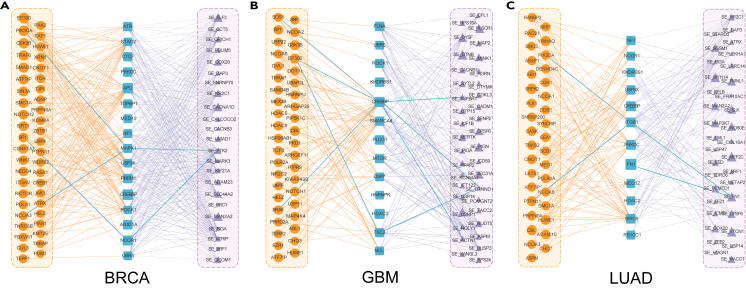
CG-SF-AS tripartite networks (A–C) The tripartite network of BRCA, GBM, and LUAD, respectively. Tripartite networks illustrate the relationships between CGs (orange circles), SFs (blue rounded rectangles), and AS events (purple triangles). The size of CG and SF nodes is proportional to the probability of being predicted as cancer genes, while the size of AS event triangles corresponds to the PSI value.

As shown in Figure 8, we constructed CG-SF-AS tripartite networks for three cancer types. These networks reveal distinct regulatory patterns between CGs, SFs, and SE events. To validate our hypothesis that the predicted cancer genes may be involved in cancer-related AS regulation pathways and play crucial roles in cancer development, we performed Kyoto Encyclopedia of Genes and Genomes (KEGG)^36^ pathway enrichment analysis on the gene sets comprising the meta-paths of CG-SF-AS within the tripartite networks. We observed that the majority of cancer-related pathways in our constructed tripartite networks encompass multiple meta-paths of AS regulation. In the BRCA case network, we observed that the predicted cancer genes PIK3CA, SP1, and NCOA3 co-regulate the PTK2 AS (SE event) through intermediate SFs MAPK1 and NCOR1 (indicated by blue edges), which are enriched on multiple cancer-related pathways in KEGG, such as the "endocrine resistance" and "choline metabolism in cancer". Interestingly, it has been demonstrated that the endocrine resistance metabolisms are closely related to the occurrence of breast cancer.^37^ Similarly, in the GBM case network (Figure 8B), we observed pathways enriched in "FoxO signaling," "adherens junction", etc. In particular, the CTNND1-related splicing regulation has been reported to promote tumor aggressiveness in epithelial-mesenchymal transition (EMT). In the LUAD case network (Figure 8C), the regulation pathways (indicated by blue edges) are enriched in lung cancer-related pathways such as "small cell lung cancer," "proteoglycans in cancer," etc. In conclusion, based on the three case studies in different cancer types, we found that our predicted cancer genes participate in cancer-related AS regulations, and the genes involved in meta-paths of AS regulation are closely related to cancer-related pathways.

## Discussion

Identifying cancer genes is crucial in cancer diagnosis and treatment. The integration of diverse multi-omics data offers a compelling opportunity for comprehensive analyses of cancer genes through computational methodologies. Despite the numerous computational methods proposed in the past decade, the quest for effective and precise cancer gene prediction remains ongoing. In this context, we present a two-stage gradual-learning method, GLIMS. This method initially predicts the candidate cancer genes by integrating multi-omics data and PPI networks via HIM-GCN and then optimizes the initial predictions by incorporating the AS-based co-splicing networks. The performance of GLIMS surpassed that of most state-of-the-art methods, underscoring its effectiveness as a computational approach in predicting cancer genes.

### Limitations of the study

While GLIMS exhibits outstanding performance in the realm of cancer gene identification, there are still some limitations that call for further improvement. These limitations primarily stem from the restricted knowledge of cancer genes within specific cancer types. To address this, future research endeavors can incorporate the principles of transfer learning, thereby enhancing predictive accuracy and generalizability. Additionally, exploring network-based methodologies to incorporate AS information more accurately could be beneficial, which would allow a more precise characterization of how cancer genes regulate cancer development at the post-transcriptional level.

## STAR★Methods

### Key resources table

**Table:** 

REAGENT or RESOURCE	SOURCE	IDENTIFIER
**Deposited data**		

Multi-omics data of cancer samples	TCGA	https://xenabrowser.net/
PPI network	PICKLE	http://www.pickle.gr/
Transcript TPMs of normal tissues	GTEx	https://www.gtexportal.org/home/

**Software and algorithms**		

Python	Python	https://www.python.org/downloads/release/python-379/
Tensorflow	GitHub	https://github.com/tensorflow/docs/tree/r1.15
SUPPA2	GitHub	https://github.com/comprna/SUPPA
GLIMS	This paper	https://github.com/sherry-0805/GLIMS/

### Resource availability

#### Lead contact

Further information and requests for raw data and code should be directed to and will be fulfilled by the lead contact, Xuequn Shang (shang@nwpu.edu.cn).

#### Materials availability

All materials reported in this paper will be shared by the lead contact upon request.

#### Data and code availability

• All datasets used in this study are publicly available and listed in the manuscript. Accession numbers are listed in the key resources table.
• The source code of the GLIMS model is available at https://github.com/sherry-0805/GLIMS, and the accession numbers are listed in the key resources table.
• Any additional information required is available by the contact upon request.

### Method details

#### Model training and parameter setting

We adopted the 3-fold cross-validation strategy throughout model evaluation, randomly partitioning the dataset into three equal parts for each iteration, with two-thirds serving as the training set and one-third as the test set to assess the predictive accuracy in cancer gene identification. Our HIM-GCN model comprises a three-layer GCN architecture where single-layer GCNs are tasked with extracting information from each type of omics data, with input dimensions equal to the omics data dimensions and output dimensions set at 50. For integrated gene representation learning, we utilized a two-layer GCN with an input dimension of 200, a hidden layer dimension of 100, and an output dimension of 1. During the training process, the Adam optimizer was employed to adjust the learning rate, starting at a baseline of 0.001. The number of training epochs was configured to 6000 for pan-cancer analysis and reduced for specific cancer gene prediction models (2000 for BRCA, 3000 for GBM, and 1500 for LUAD) to avert the risk of overfitting.

#### Collection and processing of multi-omics datasets

We obtained multi-omics cancer datasets from the UCSC Xena platform (https://xenabrowser.net/) and the TCGA data hub.^3^ To identify cancer genes, our study leveraged data on gene expression, methylation, mutation, and copy number variation. The pan-cancer multi-omics data were collected from the 'GDC Pan-cancer' Cohort, while data for the three specific cancer types, namely BRCA, GBM, and LUAD, were sourced from the 'GDC TCGA' Cohort. The gene expression data were processed using $\log_{2}\left(FPKM+1\right)$ transformation on FPKM normalized counts and incorporated expression fold-change features between normal and cancer groups. The gene methylation levels were obtained by averaging the methylation values of all CpGs on corresponding genes. For mutation data, we calculated mutation frequency features in cancer samples. To reduce the effect of data noise and data variation, we used the multi-omics data of overlapping genes found in common samples for each cancer. The human PPI network data were downloaded from PICKLE^38^ (http://www.pickle.gr/). In each cancer study, we selected the maximum connected subnetwork in PPI network based on the overlapping genes of multi-omics data to perform downstream analysis, which included 15,796 genes and 210,030 edges.

#### Generating positive and negative samples

We compiled positive cancer genes from the COSMIC database^39^ (https://cancer.sanger.ac.uk/cosmic/) and the NCG database^32^ (http://ncg.kcl.ac.uk/cancer_genes.php) for pan-cancer analyses. For analyses specific to individual cancer types, we sourced positive genes exclusively from COSMIC.^39^ The negative cancer genes are defined by excluding all cancer- and disease-related genes that were collected from multiple databases, including COSMIC,^39^ NCG,^32^ OMIM^40^ (https://omim.org/), and KEGG Cancer pathways (https://www.gsea-msigdb.org/gsea/msigdb/). Finally, a list of 2660 genes was generated to select negative samples. In pan-cancer analysis, all the genes in the negative gene list were utilized. In the specific cancer analysis, the negative samples were randomly selected from the negative gene list at a frequency tenfold that of the positive samples.

#### Collection and processing of alternative splicing data

We used SUPPA2^41^ to obtain AS events and their alternative splicing levels based on the transcript expression data of genes and human transcript annotation information. The cancer transcript expression data (TPMs) were downloaded from TCGA Pan-cancer studies using the UCSC Xena platform (https://xenabrowser.net/datapages/), and the transcript TPMs of normal tissues were downloaded from GTEx (V8)^42^ (https://www.gtexportal.org/home/). We collected AS event data in three cancer types (BRCA, GBM, and LUAD) and corresponding normal tissues. In total, seven kinds of AS events were obtained by SUPPA2,^41^ including the skipping exon (SE), mutually exclusive exons (MX), alternative 5’ splice-site (A5), alternative 3’ splice-site (A3), retained intron (RI), alternative first exon (AF), and alternative last exon (AL). In order to obtain more reliable AS information, we filtered out all AS events with the following conditions: (1) expressed on more than half of cancer samples and occurred on normal samples; (2) average PSI value > 0.1 in cancer samples; (3) absolute logarithmic fold-change > 1.5 between cancer and normal sample groups. The selected AS events in different cancers are shown in Table S1.

#### Construction of HIM-GCN model

HIM-GCN is an open hierarchical ensemble model based on GCN to integrate multi-omics data and PPI network to learn integrative representations of genes and predict cancer genes using semi-supervised learning manner. GCN is a generalized concept of graph-based convolutional network, which performs convolutional operations by layer-wise propagation rule as,

(Equation 1) $$H^{\left(l+1\right)}=\sigma \left({\tilde{D}}^{-\frac{1}{2}}\tilde{A}{\tilde{D}}^{-\frac{1}{2}}H^{\left(l\right)}W^{\left(l\right)}\right)$$

Where A is the adjacency matrix of graph G, $\tilde{A}=A+I_{N}$ is the adjacency matrix of A with added self-connections, $I_{N}$ is the identity matrix; $\tilde{D}$ is the degree matrix of $\tilde{A}$ and ${\tilde{D}}_{ii}=\Sigma_{j}{\tilde{A}}_{ij}$; $H^{\left(l\right)}\in N\times d$ is the hidden-feature matrix of nodes in the l−th layer, and $H^{\left(0\right)}=X$ is the initial feature matrix of nodes; $W^{\left(l\right)}$ is the layer-specific weight matrix; σ(·) is the activation function used for propagation. It has been proved the above graph-based propagation rule can be solved by first-order approximation of localized spectral filters on graphs.^21^

Based on four types of multi-omics data (gene expression, mutation, methylation, and copy number variation, respectively), we can derive four feature matrices, within which rows represent genes and columns represent patients. To address the high-dimensionality inherent in each feature matrix, we employed the principal component analysis (PCA) to derive low-dimensional features. Specifically, for gene expression and methylation data, we selected principal components with an explained variation ratio exceeding 0.98. In terms of mutation and copy number data, we considered principal components with a ratio surpassing 0.7. The low-dimensional features of each gene were mapped to its corresponding coding protein in PPI network and resulting in the construction of four attributed gene association networks $G_{\exp}$, $G_{mut}$, $G_{meth}$ and $G_{cnv}$.

Then, we used one-layer GCN models $Z_{t}$ to learn gene representations on each gene association network $G_{t}$ as following rules,

(Equation 2) $$Z_{\exp}=\text{ReLU}\left(\hat{A}X_{\exp}W_{\exp}^{\left(0\right)}\right)$$

(Equation 3) $$Z_{mut}=\text{ReLU}\left(\hat{A}X_{mut}W_{mut}^{\left(0\right)}\right)$$

(Equation 4) $$Z_{meth}=\text{ReLU}\left(\hat{A}X_{meth}W_{meth}^{\left(0\right)}\right)$$

(Equation 5) $$Z_{cnv}=\text{ReLU}\left(\hat{A}X_{cnv}W_{cnv}^{\left(0\right)}\right)$$

Where $\hat{A}={\tilde{D}}^{-\frac{1}{2}}\tilde{A}{\tilde{D}}^{-\frac{1}{2}}$, $X_{t}$ is the initial feature matrix of genes in data t, $W_{t}^{\left(0\right)}$ is the weight matrix in the first layer.

With the output representations of four GCN models, we further designed a two-layer GCN model to learn the integrative representations of genes by concatenating all types of representations and constructing an integration gene association network. The integrative GCN model $Z_{\mathrm{int}}$ was implemented in the form as follows,

(Equation 6) $$Z_{\mathrm{int}}=\text{Softmax}\left(\hat{A}\text{ReLu}\left(\hat{A}X_{\mathrm{int}}W_{\mathrm{int}}^{\left(0\right)}\right)W_{\mathrm{int}}^{\left(1\right)}\right)$$

Where $\hat{A}={\tilde{D}}^{-\frac{1}{2}}\tilde{A}{\tilde{D}}^{-\frac{1}{2}}$ , $X_{\mathrm{int}}=Z_{\exp}⊕Z_{mut}⊕Z_{meth}⊕Z_{cnv}$ is the integration feature matrix obtained by directly concatenating the multi-omics representation matrices, $W_{\mathrm{int}}^{\left(0\right)}$ and $W_{\mathrm{int}}^{\left(1\right)}$ represent the initial weight matrix and weight matrix of the first layer in this model.

In order to consider the issue of extreme imbalance of positive and negative samples, we designed a weighted cross-entropy loss function to optimize the HIM-GCN model,

(Equation 7) $$\text{L}\left(\Theta \right)=-\frac{1}{N}\sum_{i=1}^{N}\left\{\alpha_{1}ylog\left(Z\left(W,X\right)\right)+\alpha_{2}ylog\left(1-Z\left(W,X\right)\right)\right\}+\lambda_{1}\left\|W{\|}_{1}+\lambda_{2}\right\|W{\|}_{2}$$

Where $\alpha_{1}=N/N_{positive}$, $\alpha_{2}=N/N_{negative}$; $N_{positive}$ and $N_{negative}$ are the numbers of positive and negative samples respectively; N is the total number of labeled samples in the training data; y∈{0,1} is the binary label of sample; W represents the learned weight matrix; X is the feature matrix of multi-omics data; $\lambda_{1}$ and $\lambda_{2}$ are the L1 and L2 regularization parameters to reduce the risk of overfitting.

#### Optimization of initial prediction using co-splicing network

We defined those genes as predicted cancer genes if their probability of being positive, as determined by HIM-GCN, exceeded 0.7. Furthermore, we identified informative AS events through differential analysis and considered these to be cancer-related AS events. To construct the cancer-related co-splicing network between cancer genes and AS events, we calculated the relationships between them by utilizing the partial correlation calculation method,^43^ which considers the effect of global correlations between the focus genes and AS located genes. Given a pair of focus gene $g_{i}$ and AS event $e_{j}$, which belongs to gene $g_{j}$, the partial correlation between gene $g_{i}$ and AS event $e_{j}$ is defined as,

(Equation 8) $$r_{g_{i}e_{j}|g_{j}}=\frac{r_{g_{i}e_{j}}-r_{g_{j}e_{j}}r_{g_{i}g_{j}}}{\sqrt{\left(1-r_{g_{j}e_{j}}^{2}\right)\left(1-r_{g_{i}g_{j}}^{2}\right)}}$$

Where $r_{g_{i}e_{j}}$ is the Pearson correlation coefficient of gene $g_{i}$ and AS event $e_{j}$ based on co-expression. Similarly, $r_{g_{j}e_{j}}$ is correlation coefficient between $g_{j}$ and $e_{j}$, $r_{g_{i}g_{j}}$ is the correlation coefficient between $g_{i}$ and $g_{j}$. $r_{g_{i}e_{j}|g_{j}}$ is the partial correlation coefficient between $g_{i}$ and $e_{j}$ under the condition that $e_{j}$ is associated with gene $g_{j}$. We calculated the partial correlation coefficients of all pairs between the predicted cancer genes and cancer-related AS events and reserved the high-confident relationships to construct the cancer-related co-splicing network. In order to determine a reasonable cutoff parameter in the selection of high-confident relationships, we randomly shuffled both gene and AS events five times and flexibly defined the cutoff scores by controlling the permutation version fdr at a reasonable level (fdr < 0.05).

Based on the cancer-related co-splicing network, we deemed that two genes tend to have stronger co-regulatory relationship if they regulate more common cancer-related AS events in the co-splicing network. Therefore, we constructed the co-regulation network of cancer genes by analyzing their common AS targets in cancer-related co-splicing network. Given a pair of cancer genes $g_{i}$ and $g_{j}$ in the co-splicing network, the co-regulation weight between them is defined as,

(Equation 9) $$W\left(g_{i},g_{j}\right)=\frac{2\left|T\left(g_{i}\right)\cap T\left(g_{j}\right)\right|}{\left|T\left(g_{i}\right)\right|+\left|T\left(g_{j}\right)\right|}$$

Where $T\left(g_{i}\right)$ is the AS target set of $g_{i}$ in a cancer-related co-splicing network. We calculated the weight for each pair of initial candidate cancer genes and constructed a co-regulation network by requiring that survival edges have higher weights (default threshold ≥ 0.3). The cancer genes co-regulation network provides the global cooperation map of cancer genes in the regulation of cancer-related alternative splicing.

We adopted the PageRank^44^ algorithm to prioritize all initial predicted cancer genes based on the co-regulation network. PageRank algorithm is initially designed to work on the directed network, the undirected co-regulation network of cancer genes was transformed to a directed network in which each edge has two directions between the start and end nodes. Specifically, PageRank is a network propagation algorithm using the random walk with restart strategy to predict the importance of nodes in a directed hyperlink network. The main process of propagation can be represented as,

(Equation 10) $$p_{k+1}=\alpha p_{0}+\left(1-\alpha \right)Wp_{k}$$

Where $p_{0}\in R^{N}$ represents the importance vector of initial prior information of nodes, $p_{k}\in R^{N}$ represents the importance vector at the step k in the network propagation process. $W=AD^{-1}$, A is the adjacency matrix of the network and D is the diagonal degree matrix of the network, in which the diagonal elements are the out degrees of corresponding nodes, all other elements are 0; α is the trade-off parameter to consider the prior information and network smoothing in the whole process of information propagation.

In order to evaluate the performance of prediction results before and after co-regulation network optimization, we employed the partial ROC measure. The genes included in the candidate cancer genes and cancer driver genes of NCG were labeled as positives, and the other genes were labeled as negatives.^32^^,^^39^^,^^45^ Partial ROC measure $AUC_{n}$^11^ considers the number of positives with predicted ranks higher than the n th highest-ranked negative, assessed for all values from 1 to n. It is defined as,

(Equation 11) $$AUC_{n}=\frac{1}{nT}\sum_{i=1}^{n}T_{i}$$

Where T is the total number of positives and $T_{i}$ denotes the number of positives higher than the i th highest-ranked negative.

## Supplemental nformation

Table S1. The selected AS events in different cancer types, related to Figure 1
